# Polyphasic characterization and genetic relatedness of low-virulence and virulent *Listeria monocytogenes* isolates

**DOI:** 10.1186/1471-2180-12-304

**Published:** 2012-12-26

**Authors:** Sylvie M Roche, Olivier Grépinet, Annaëlle Kerouanton, Marie Ragon, Alexandre Leclercq, Stéphanie Témoin, Brigitte Schaeffer, Gilbert Skorski, Laurent Mereghetti, Alban Le Monnier, Philippe Velge

**Affiliations:** 1 INRA, UR 1282 Infectiologie Animale et Santé Publique, Agents transmissibles et Infectiologie, F-37380, Nouzilly and IFR, 136, France; 2 Université de Tours, UMR1282 Infectiologie et Santé Publique, F-37000, Tours, France; 3 ANSES LERQAP, Unité Caractérisation et Epidémiologie Bactérienne, F-94706, Maisons-Alfort, France; 4 Institut Pasteur, French National Reference Center and WHO collaborating Center for Listeria, F-75724, Paris, France; 5 INRA, UR 0341 Mathématiques et Informatique Appliquées, F-78352, Jouy-en-Josas, France; 6 PHYLOGENE, F-30620, Bernis, France; 7 Université François Rabelais de Tours, EA3854 « Bactéries et risque materno-fœtal », Tours, France and CHRU, F-37044, Tours, France; 8 Current address: ANSES UHQPAP, Unité Hygiène et Qualité des Produits Avicoles et Porcins, F-22440, Ploufragan, France; 9 Current address: Université Paris Sud Orsay, CNRS-UMR807, Unité Ecologie Systématique Evolution, F-91400, Orsay, France; 10 Current address: Institut Pasteur, Centre de Ressources Biologiques de l’Institut Pasteur (CRBIP), F-75724, Paris, France; 11 Current address: Laboratoire de Microbiologie-Hygiène, Centre Hospitalier de Versailles, Université Paris Sud, F-78150, Le Chesnay, France

## Abstract

**Background:**

Currently, food regulatory authorities consider all *Listeria monocytogenes* isolates as equally virulent. However, an increasing number of studies demonstrate extensive variations in virulence and pathogenicity of *L. monocytogenes* strains. Up to now, there is no comprehensive overview of the population genetic structure of *L. monocytogenes* taking into account virulence level. We have previously demonstrated that different low-virulence strains exhibit the same mutations in virulence genes suggesting that they could have common evolutionary pathways. New low-virulence strains were identified and assigned to phenotypic and genotypic Groups using cluster analysis. Pulsed-field gel electrophoresis, virulence gene sequencing and multi-locus sequence typing analyses were performed to study the genetic relatedness and the population structure between the studied low-virulence isolates and virulent strains.

**Results:**

These methods showed that low-virulence strains are widely distributed in the two major lineages, but some are also clustered according to their genetic mutations. These analyses showed that low-virulence strains initially grouped according to their lineage, then to their serotypes and after which, they lost their virulence suggesting a relatively recent emergence.

**Conclusions:**

Loss of virulence in lineage II strains was related to point mutation in a few virulence genes (*prfA, inlA, inlB, plcA*). These strains thus form a tightly clustered, monophyletic group with limited diversity. In contrast, low-virulence strains of lineage I were more dispersed among the virulence strains and the origin of their loss of virulence has not been identified yet, even if some strains exhibited different mutations in *prfA* or *inlA*.

## Background

*Listeria monocytogenes*, a facultative intracellular pathogen, is one of the major causes of food-borne infection in humans [1]. Although rare, invasive listeriosis is a public health concern due mainly to its high fatality rate evaluated at 20-30% [2]. The clinical outcome of listeriosis is influenced by the pathogenic potential of the infecting strain which is in part related to its serotype [3]. It is now known that isolates 1/2a, 1/2b and 4b are responsible for 96% of human infections and most outbreaks are caused by strains of serotype 4b whereas serotype 1/2a has been associated with sporadic cases [4]. Serotypes 4a and 4c are predominant in animal, food or environment [5].

Unfortunately, there is currently no standard definition of virulence levels and no comprehensive overview of the evolution of *L. monocytogenes* strains taking into account the presence of low-virulence strains [5]. Different studies have shown that *L. monocytogenes* isolates form a structured population, composed of divergent lineages [6]. The large majority of isolates clusters into two lineages, but two additional lineages have been identified. However, these lineages correspond more to different but overlapping niches than to virulence-related clusters. We previously described low-virulence *L. monocytogenes* strains using a method that combines a plaque-forming (PF) assay with the subcutaneous (s.c.) inoculation of mice [3]. Using the results of cell infection assays and phospholipase activities, the low-virulence strains were assigned to one of four groups by cluster analysis. Sequencing of virulence-related genes highlighted the molecular causes of low virulence. Group I included strains that exhibited two different types of mutation in the *prfA* gene: either a single amino acid substitution, PrfAK220T, or a truncated PrfA, PrfAΔ174-237 [7]. In Group III, strains exhibited the same mutations in the *plcA*, *inlA* and *inlB* genes that lead to a lack of InlA protein, an absence of PI-PLC activity and a mutated InlB [8]. The fact that numerous strains exhibit the same substitutions in virulence genes suggests that they could have common evolutionary pathways. In contrast, Ragon *et al.* reported that numerous *L. monocytogenes* strains exhibit different mutations in the *inlA* gene due to convergent evolution [9]. These data emphasize the interest of providing a framework for the population study based on the virulence of this bacterium.

The aim of this study was to assign the new low-virulence strains identified by different methods to phenotypic and genotypic Groups using cluster analysis, and to study their relatedness with virulent *Listeria monocytogenes* strains using pulsed-field gel electrophoresis and multi-locus sequence typing analyses

## Results

### Phenotypic characterisation of the low-virulence strains

The combination of PF assays followed by s.c. injections of immunocompetent mice, allowed us through different studies, to collect 43 low-virulence strains mainly of serotypes 1/2a (51%) and 4b (28%), which are usually related to sporadic and epidemic human cases of listeriosis, respectively [4] (Table 1). In this study, a strain is considered a low-virulence strain when fewer than 4 mice out of 5 inoculated become infected with a mean number of bacteria in the spleen less than 3.45 ± 0.77 log [3].

**Table 1:** **Characterization of the low-virulence *L. monocytogenes *strains**

**Strains**	**Sub-cutaneous test**		**Phenotypic Group**^**c**^	**Mutations**	**Genotypic Group**^**d**^	**MLST**	**PFGE types**	
	**Mean (log spleens) ± S.D.**^**a**^	**I/T**^**b**^				**Sequence types**	**ApaI**	**AscI**
CHU 860776 ^e^	0	0/5	I	PrfA K220T, truncated InlA (76 AA)	Ia	13	2	2
CNL 895803 ^e^	0	0/5	I	PrfA K220T, truncated InlA (76 AA)	Ia	13	2	2
CNL 895804 ^e^	0	0/5	I	PrfA K220T, truncated InlA (76 AA)	Ia	13	2	2
CNL 895806 ^e^	0	0/5	I	PrfA K220T, truncated InlA (76 AA)	Ia	13	2	2
CNL 895809 ^e^	0	0/5	I	PrfA K220T, truncated InlA (76 AA)	Ia	13	2a	2
CNL 895793 ^e^	0	0/5	I	PrfA K220T, truncated InlA (76 AA)	Ia	13	2a	2
SO49 ^e^	0	0/5	I	PrfA K220T, truncated InlA (76 AA)	Ia	13	3	23
AF10 ^e^	0	0/5	I	PrfA K220T, truncated InlA (76 AA)	Ia	13	3	23
99EB24LM	0	0/5	I	PrfA K220T, truncated InlA (76 AA)	Ia	13	3	23
99EB04LM	0	0/5	I	PrfA K220T, truncated InlA (76 AA)	Ia	13	3	23
BO18 ^e^	1.31	1/5	I	PrfAΔ174-237, truncated InlA (188 AA)	Ib	31	77a	61b
BO38 ^e^	0	0/5	I	PrfAΔ174-237, truncated InlA (188 AA)	Ib	31	77a	61b
AF95 ^e^	0	0/5	I	PrfAΔ174-237, truncated InlA (188 AA)	Ib	31	77a	61c
99EB15LM	0	0/5	I	PrfAΔ174-237, truncated InlA (188 AA)	Ib	31	21a	20
NP 26	0	0/5	I	PrfA K130Q	Ic	2	61a	3
454 ^e^	3.26 ± 0.53	3/20	II	mutated PC-PLC (D61E, L183F, Q126K, A223V)		10	9	11
CNL 895807 ^e^	3	1/25	III	truncated InlA (25 AA), mutated InlB (A117T, V132I), PI-PLC T262A	IIIa	193	1	1
416 ^e^	0	0/5	III	truncated InlA (25 AA), mutated InlB (A117T, V132I), PI-PLC T262A	IIIa	193	1	1
417 ^e^	2.81 ± 1.47	2/20	III	truncated InlA (25 AA), mutated InlB (A117T, V132I), PI-PLC T262A	IIIa	193	1	1
BO43 ^e^	2.53	1/5	III	truncated InlA (25 AA), mutated InlB (A117T, V132I), PI-PLC T262A	IIIa	193	1a	1a
CNL 895795 ^e^	0	0/5	III	truncated InlA (25 AA), mutated InlB (A117T, V132I), PI-PLC T262A	IIIa	193	1a	1a
DSS794AA1	0	0/5	III	truncated InlA (25 AA), mutated InlB (A117T, V132I), PI-PLC T262A	IIIa	193	144	33a
DSS1130BFA2	0.47	1/5	III	truncated InlA (25 AA), mutated InlB (A117T, V132I), PI-PLC T262A	IIIa	193	143	129
DPF234HG2	2.76 ± 0.04	2/5	III	truncated InlA (25 AA), mutated InlB (A117T, V132I), PI-PLC T262A	IIIa	193	145	33b
AF105 ^e^	0	0/5	III	truncated InlA (576 AA)	IIIb	9	81	64
442 ^e^	0	0/5	IV			1	6	7
02-99 SLQ 10c Al	2.9 ± 0.05	2/5	IV			1	11	7
3876	3.42 ± 0.2	3/5	IV			1	142	113
3877	2.7 ± 0.2	3/5	IV			1	142	113
N2	3.59 ± 0.48	2/5	IV			10	11	4b
CR282 ^e^	3.01 ± 0.61	2/10	IV			195	158	85
LSEA 99–23 ^f^	4.49 ± 0.89	3/5	IV	truncated InlA (576 AA)		9	21a	20
LSEA 99-4^f^	3.67 ± 0.81	3/5	IV			198	48	101
09-98 SRV 10a Al1	0	0/5	IV			4	37	38b
449 ^e^	0	0/5	V	3 AA deletion at position 742 in InlA		194	8	6
BO34 ^e^	3.63 ± 0.56	5/10	V			2	4a	3
464 ^e^	2.59 ± 0.39	9/15	V			1	9c	4a
09-98 SRV 10b Al2	3.54 ± 0.27	3/5	V			54	135	124
11-99 SRV 1a Al	0	0/5	V			4	37	38b
09-98 HPR 50a Al1	0	0/5	V	3 AA deletion at position 742 in InlA		6	67a	98a
436 ^e^	2.81 ± 0.68	12/20	VI			2	4	3
LSEA 00–14 ^f^	0	0/5	VI			2	106	3a
04-99 EBS 1 lb Al	2.53 ± 1.76	2/5	VI			54	139	125

^a^ Log numbers of *Listeria* recovered from spleens three days after sub-cutaneous injection into the left hind footpads of immunocompetent Swiss mice with 10^4^ CFU in 50 μL. Values are from infected mice.

^b^ Ratio of infected mice to inoculated mice in sub-cutaneous test.

^c^ These groups are based on combined values of cell invasion, plaque formation, and phospholipase activities.

^d^ These groups are based on sequencing of *prfA*, *plcA*, *plcB*, *inlA* and *inlB* genes.

^e^ Results already published in Roche *et al*. [7].

^f^ Results already published in Kerouanton *et al. *[10].

As previously performed, these low-virulence strains were classified using an ascendant clustering hierarchical technique [3]. Six groups according to the values of four factors (level of cell invasion, number of plaques formed, and enzymatic activities of the two phospholipases C) have been obtained (Table 1). Group-I included 15 strains that did not enter cells, formed no plaques and had no phospholipase activity. Group-II consisted of only one strain entering cells, forming no plaques and only expressing PI-PLC activity. Group-III comprised nine strains entering cells, forming no plaques and only expressing PC-PLC activity. In this new analysis, the previously described Group-IV [7] has now been divided into 3 sub-Groups. The new Group-IV included nine strains forming plaques but fewer than virulent strains (mean 3 log versus 5). Three out of 9 strains were also characterized by a very low level of PC- and PI-PLC. The new Group-V comprised six strains also forming plaques but fewer than virulent strains and characterized by their very high PI-PLC activity. Finally, Group-VI contained three strains forming plaques within 48 h. In contrast the other strains formed plaques within 24 h, classic time necessary to count the plaque number.

### Genotypic characterisation of the low-virulence strains

Sequencing the *prfA*, *plcA*, *plcB*, *inlA* and *inlB* genes allowed us to observe that some phenotypes correlate with genotypic mutations which have been demonstrated to be the cause of the low virulence (Table 1) [7]. The sequences of the PrfA, InlA and ActA fragment were compared to those of the EGDe strain (serotype 1/2 - GenBank accession number AL591824) or F2365 strain (serotype 4 - GenBank accession number AE017262), according to the serotypes of the strains.

The phenotypic Group-I strains exhibited mutations in PrfA compared to the EGDe strain and were subdivised into 2 genotypic Groups: the PrfAK220T (genotypic Group-Ia) and the truncated PrfAΔ174-237 (genotypic Group-Ib) previously described [8,11]. One strain (NP26) exhibited a new putative causal mutation in *prfA*, K130Q, and is the only one of serotype 4b exhibiting a PrfA mutation (herein defined as genotypic Group-Ic).

Two genotypic Groups were also identified for the phenotypic Group-III strains. One harbored exactly the same mutations in the *plcA*, *inlA* and *inlB* genes, characteristic of the previously genotypic Group-IIIa [8]. Only one strain (AF105) belonged to Group-IIIb and harbored a mutation at least in the *inlA* gene.

No genotyping Group has been defined for the phenotypic Groups-II because this Group is formed by only one strain. The Group-IV, -V and –VI strains did not exhibit specific DNA sequence of the *prfA*, *inlA* and *actA* fragment genes, that allowed us to assign genotyping Groups. No causal mutations could have been displayed explaining the low virulence of these Groups.

### PFGE profiles

To study the genetic relationships between the low-virulence strains, the 43 low-virulence strains were compared with 49 virulent strains (based on both the mouse s.c. inoculation and PF assays) selected on the basis of matching serotypes and origins (Additional file 1).

**Figure 1:**
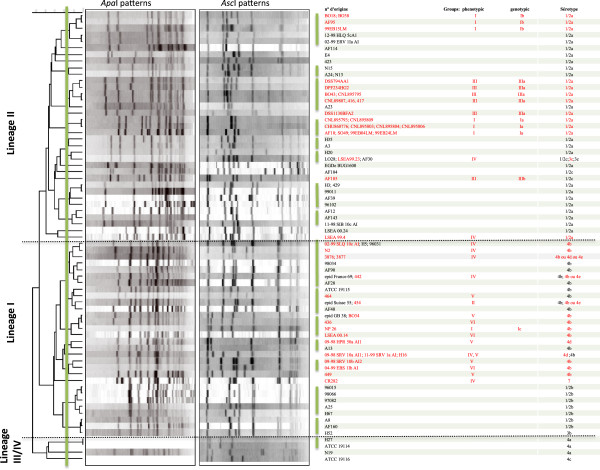
**Dendrogram constructed for PFGE analysis using the UPGMA method with BioNumerics v.4.6 software showing the genetic relationships between 92** ***L. monocytogenes *strains.** The low-virulence strains are in red. Green lines indicate the division into clusters of strains having 85% similarity. Phenotypic groups were based on results of cellular entry, plaque formation, and the two phospholipase C activities. Genotypic Groups were defined as follows: Group-Ib included the strains with PrfAK220T. Group-Ia included the strains with PrfAΔ174-237. Group-IIIa had the same mutations in the *plcA*, *inlA* and *inlB* genes. Group-Ic showed the K130Q mutation.

This analysis revealed three major branches (Figure 1) probably corresponding to the lineages I, II and IV described by Ward *et al. by* a SNP analysis [12]. In their study lineages I and III isolates formed, indeed, a sister group to lineage II strains, while the lineage IV represented a divergent sister clade. However, the small number of lineage IV strains did not allow us to conclude in this distribution. Nonetheless, as observed by Ward *et al*., lineage I included strains of serotype 1/2b, 4b, 4d, 4e, 3b and 7, whereas lineage II included strains of serotype 1/2a, 1/2c and 3a. Lineage III and IV included strains of serotype 4a, 4b and 4c. PFGE typing of the 92 isolates resulted in 69 different patterns, most of them grouped into 16 clusters with a similarity percentage above 85%. All strains gave interpretable PFGE patterns after restriction by *Asc*I enzyme, whereas three virulent strains of lineage III/IV (serotype 4a and 4c) gave no profiles after *Apa*I restriction, possibly due to the methylation of restriction sites [13,14].

No clear correlation could be made between the PFGE clusters and the virulence levels of the strains and even though seven clusters included only virulent strains, the low-virulence strains were distributed in 9 clusters out of 16 (indicated by green lines in Figure 1), often mixed with virulent strains. Within the same lineage, the low-virulence strains were clustered according to their serotype. This observation is supported by the fact that strain NP26 belongs to the phenotypic Group-I which was grouped in lineage I with serotype 4b strains, whereas all the other strains of the phenotypic Group-I were grouped in lineage II with serotype 1/2a strains.

In the lineage II, the low-virulence strains were grouped according to their genotyping Groups, but were sometimes clustered with virulent strains. Only strains of the genotypic Group-Ia formed one specific cluster. All strains of the genotypic Group-IIIa were grouped together, but on the same branch as strain A23 (similarity percentage >80%). This clustering can be explained by the demonstration that the A23 strain had the same genotypic mutations as the Group-IIIa strains, but exhibited some virulence in our *in vivo* and *in vitro* virulence tests [15]. In the same way, all strains of the genotypic Group-Ib belonged to the same cluster, but with two virulent strains.

In the lineage I, the phenotypic Groups-IV, -V and -VI did not form specific clusters but were mixed with virulent strains (Figure 1). This is probably related to the absence of a genotypic Group and probably corresponds to multiple genomic backgrounds. No low-virulence strain was found in lineage III/IV, but the small number of strains in this lineage hampered us to conclude in the rate of low-virulence strains.

### Sequencing of virulence and housekeeping genes

**Figure 2:**
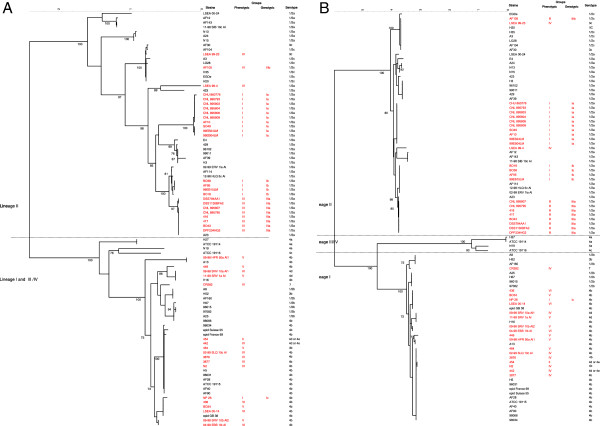
**A Dendrogram of the *prfA*, *actA *and *inlA *gene sequencing using the NJ method with BioNumerics v.4.6 software showing the genetic relationships between 92** ***L. monocytogenes *strains.** The tree was constructed on the basis of the mean matrix distances of the three virulence genes. The low-virulence strains are in red. Phenotypic groups were based on results of cellular entry, plaque formation, and the two phospholipase C activities. Genotypic groups were defined as follows: Group-Ib included the strains with PrfAK220T, Group-Ia included the strains with PrfAΔ174-237, and Group-IIIa had the same mutations in the *plcA*, *inlA* and *inlB* genes. Group-Ic showed the K130Q mutation. **B**. MLST-based dendrogram using the NJ method with BioNumerics v4.6 software showing the genetic relationships between 92 *L. monocytogenes* strains. The tree was constructed on the basis of the mean matrix distances of seven housekeeping genes (*acbZ*, *bglA*, *cat*, *dapE*, *dat*, *ldh*, and *lhkA*). The low-virulence strains are in red.

To investigate the population structure and diversity of the low-virulence strains compared to virulent strains, three virulence genes were sequenced (*prfA*, *inlA* and *actA*) as well as seven housekeeping genes (*acbZ*, *bglA*, *cat*, *dapE*, *dat*, *ldh*, and *lhkA*). The dendrograms of the concatenated nucleotide sequences of virulence and housekeeping genes performed with the NJ method were presented Figure 2A and 2B, respectively. They showed different relationships among lineages and in part for some lineage I low-virulence strains. In the housekeeping-gene tree, lineage III/IV strains formed a sister group to lineage I isolates as previously described [16]. However, as also observed by Tsai *et al.*[16], this was not the case with the virulence-gene tree where the strains of serotype 4a and 4c formed different branches. In the same way, all strains of serotype 4b were on the same branch in the housekeeping-gene tree. That was not the case in the virulence-gene tree where few strains of serotype 4b were on the same branch as strains of serotype 1/2b and 3b. Similar variations were observed for strains of serotype 1/2a which were on the same branch in the housekeeping-gene tree, whereas with the virulence-gene tree, 7 strains were on different branches than the other 34 serotype 1/2a strains (bootstrap 100%). This observation comforted the hypothesis that numerous recombinations have occurred with the virulence genes.

Similar variations between the two trees were also observed for low-virulence strains of lineage I. For example, with the virulence-gene tree 2 low-virulence strains of serotype 4b and 2 of serotype 4d were on the same branch as virulent strains of serotype 1/2b, 3b, and 7. This is not the case for the housekeeping-gene tree. As observed with PFGE, for the lineage II, both trees suggested that i) all the low-virulence strains of the same genotyping Group are on the same branch, and ii) the genotypic Group-Ia was closer to the genotypic Group-IIIa than to the genotypic Group-Ib. In lineage I, the low-virulence strains of phenotypic Groups-IV, -V and -VI were, in contrast, mixed with virulent strains showing that evolution of their virulence genes had occurred independently. This is also related to the fact that no genotyping group has been detected for these lineage I strains.

Twenty-six out of the 43 low-virulence strains (60%) and 11 out of the 49 virulent strains (22%) had a truncated InlA protein (Table 2), grouped in only 7 ST. Remarkably, all low-virulence strains of lineage II had a truncated InlA protein, compared to only three out of 18 low-virulence strains of lineage I. In addition, a correlation exists between the genotyping Groups and *inlA* mutations. All strains of the genotypic Group-Ia harboring the PrfAK220T mutation exhibited the *inlA* mutation at codon 77. Similarly, all strains of the genotypic Group-Ib harboring the PrfAΔ174-237 mutation exhibited a stop-codon at codon 189, and all strains of genotypic Group-IIIa had an insertion after the codon 13, leading to a truncated InlA.

**Table 2:** **Mutational events in the *inlA *gene**

**Sequence types (n**^**a**^**)**	**Number of strains and level of virulence**^**b**^	**Serotype**	**Genotypic Group**	***inlA***		**Location of premature stop codon**^**c**^	**Mutation**
				**Nucleotide**	**Event**		**Types**^**d**^
31 (n = 8)	4 LV	1/2a	Ib	564	C-to-T transition	189	5
	4 V	1/2a		12	deletion 1 nt	9	4
13 (n = 11)	11 LV	1/2a	Ia	228	C-to-T transition	77	15
193 (n = 8)	8 LV	1/2a	IIIa	13	insertion 1 nt	26	-
196 (n = 1)	1 V	1/2a		13	insertion 1 nt	26	-
9 (n = 8)	2 LV; 2 V	1/2c; 3c; 1/2a	IIIb	1636	deletion 1 nt	577	12
	2 V	1/2c; 3c		2053	G-to-A transition	685	11
	1 V	1/2a		1614	C-to-T transition	539	14
6 (n = 2)	1 V	4b		2219	deletion 9 nt	-	-
194 (n = 1)	1 V	4b		2219	deletion 9 nt	-	-

^a^ Number of strains in the sequence types.

^b^ Number of strains with the *inlA* event and level of virulence: V (virulent) or LV (low-virulence).

^c^ Numbers represent the amino acid position of each respective premature stop codon in InlA. The deletion of 9 nucleotides for the 2 last ST did not generate any premature stop codon.

^d^ Mutation types according to Van Stelten *et al.*[17].

### MSTree analysis

To analyze in greater detail the population structure of the low-virulence strains, the 92 strains were analyzed and compared with the 656 *L. monocytogenes* isolates included in a previous study [18]. As no low-virulence strain was found in lineage III/IV, we presented only the lineages I and II.

**Figure 3:**
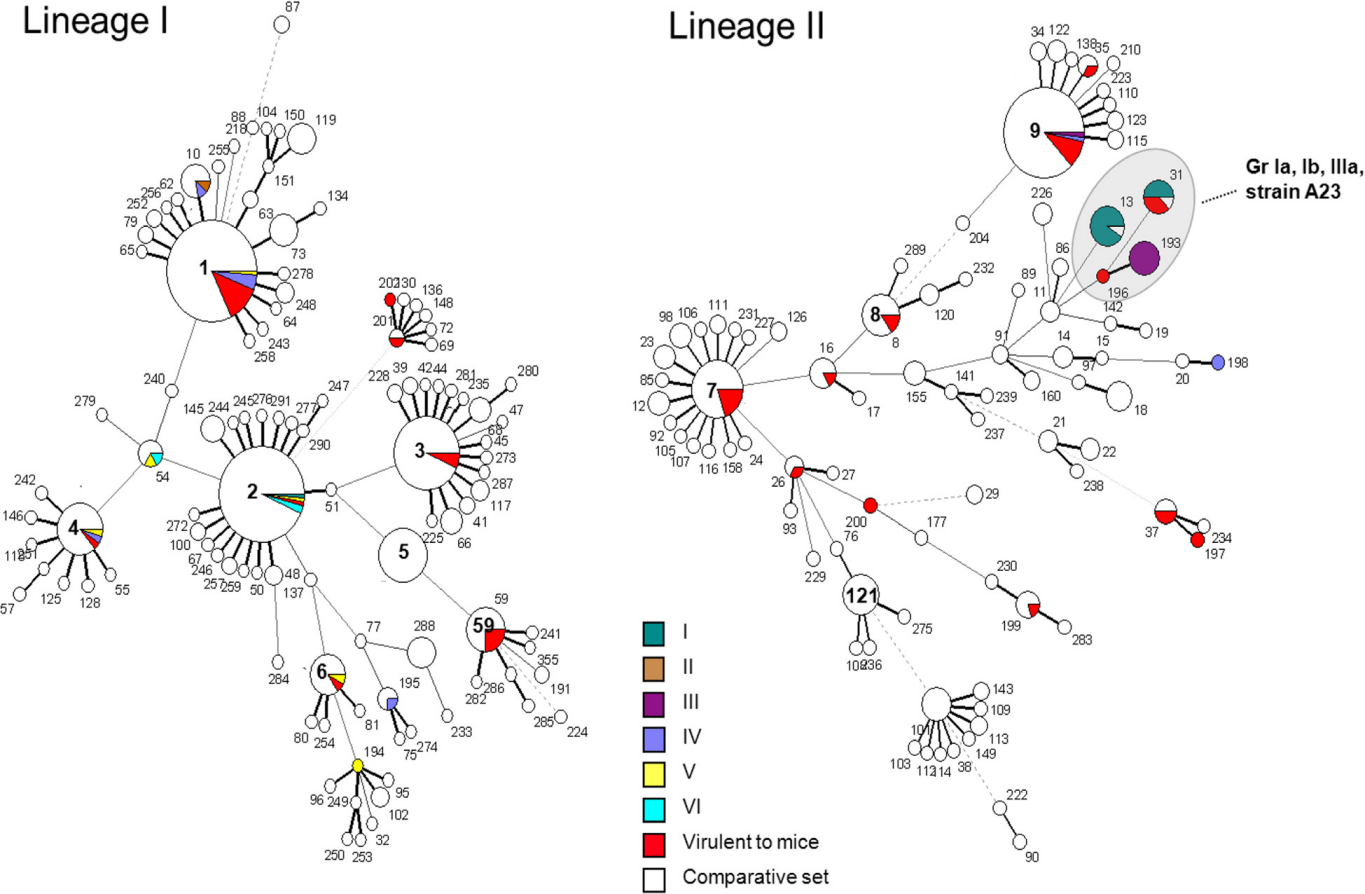
**Minimum spanning tree based on allelic profiles by using BioNumerics version 4.6.** (Applied-Maths, Sint-Martens-Latem, Belgium). The comparative set included 656 *L. monocytogenes* strains from the French Reference Centre for *Listeria* and the WHO Collaborative Centre for Foodborne Listeriosis. The experimental set included 92 *L. monocytogenes* strains defined as virulent (“virulent to mice”) or low-virulence (phenotypic Groups “I to VI”) using a virulence test combining a PF assay in HT-29 cells and sub-cutaneous inoculation of mice. Each circle corresponds to a sequence type (ST). ST numbers are given inside the circles. The lines between STs show inferred phylogenetic relationships and are represented by bold, continuous, dotted and pale dotted lines according to the number of allelic mismatches between profiles (1, 2, 3 and 4 or more, respectively); the discontinuous links are only indicative, as alternative links of equal weight may exist. Phenotypic Groups (I to VI) of low-virulence and virulent *L. monocytogenes* strains are marked in color. The comparative set of *L. monocytogenes* strains are in white. Specific STs for Groups-Ia, -Ib and -IIIa and A23 strains are in an area shaded grey.

This analysis showed that low-virulence strains of genotypic Group-Ia, -Ib, and -IIIa were distributed among three specific closely related STs (13, 31, 193) (Figure 3). The ST 13 was formed with 10 Group-Ia low-virulence strains and one strain (Lm74905) belonging to the comparative set (in white). The analysis of this strain revealed that it exhibited the PrfAK220T mutation and the same truncated InlA characterizing the genotypic Group-Ia. Likewise, the Lm85820 strain which grouped in the ST31 (in white) exhibited the same mutation in InlA than the low-virulence strains of this ST, but no mutation in PfrA. Remarkably, although all strains of the ST31 had InlA mutations, only half of these strains also had the PrfAΔ174-237 mutation. In this analysis, the A23 strain corresponds to a singleton (ST196) with only one mismatch with Group-IIIa and two with Group-Ia. It is related to Group-Ib through ST11.

Overall, half of the low-virulence strains (22 out of 43), belonging to the genotyping Groups-Ia, -Ib and -IIIa, are likely to have descended from a single virulent 1/2a ancestral bacterium. In contrast, the other strains were distributed into five clonal complexes and 10 STs and may be regarded as virulence variants of *L. monocytogenes* strains.

### Contribution of the optical mapping

**Figure 4:**
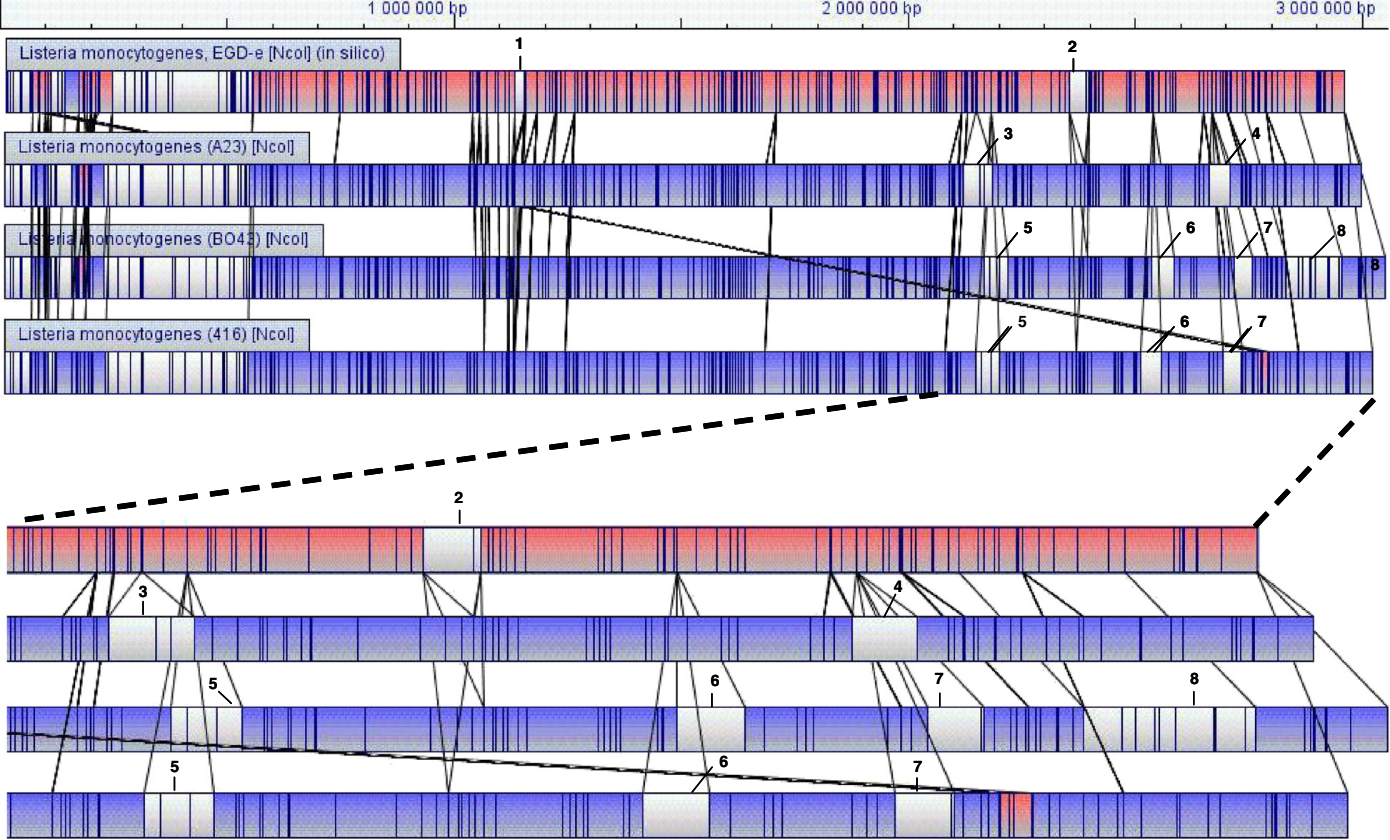
**Aligned optical maps for Group-III (BO34, 416) and A23 strains and *in silico *reference EGDe map.** In the pair-wise alignments, lines connecting two chromosomal maps indicate a discontinuity in the alignment of fragments. Chromosomal inversions are indicated by crossed alignment lines between paired maps and are highlighted in pink. Unaligned restriction fragments, representing differences between two aligned chromosomes, are shown in white; blue indicates aligned restriction fragments. Fragments 3 and 4 represent inserted fragments in the A23 chromosome. Fragments 5, 6 and 7 represent inserted fragments in the chromosomes of the BO43 and 416 strains. A supplementary fragment 8 is inserted in the chromosome of the BO43 strain.

To investigate the genomic relationship between the A23 strain and the closely related low-virulence strains belonging to Group-IIIa strains, two strains (BO43 and 416) were compared with the A23 strain using optical mapping and the *in silico* reference EGDe map (Figure 4). The EGDe optical map was approximately 20% different from the maps of the Group-IIIa and A23 strains, whereas the A23 strain showed 99% similarities with Group-IIIa. Two fragments (3 and 4) (63 and 47 kb, respectively) had been inserted in the chromosome of the A23 strain but not in the EGDe strain. Fragments 5, 6 and 7 (52, 50 and 41 kb, respectively) represent the fragments inserted in the chromosomes of the BO43 and 416 strains. A supplementary fragment 8 (125 kb) was inserted in the chromosome of the BO43 strain.

This analysis confirms that all the Group-IIIa strains are very similar to each other and to the A23 strain. Indeed the insertion of the fragment 4 is located at the same place as the fragment 7 and could be inserted in the region of the lmo2589 gene annotated as similar to a transcription regulator T and R / AcrR family. The fragment 3 present in the A23 strain is different from the fragment 5, present in the Group III strains and could explain the increase of virulence of the A23 strain. The fragment 3 could be inserted in the region of the lmo2073 gene annotated as similar to ABC transporter and the region of the lmo2074 gene (similar to unknown proteins). The fragment 5 could be inserted in the region of the lmo2105 gene, annotated as similar to ferrous iron transport protein B. The fragment 6 present in the Group III strains could explain the decrease of virulence of these strains compared to the A23 strain. Indeed the annotation of the EGDe strain indicates that this insertion was found in the lmo2467 gene, located upstream of the *clpP* gene and its promoter, involved in the rapid and adaptive response of intracellular pathogens during the infectious process [19].

## Discussion

For a long time, all *L. monocytogenes* isolates were regarded as strictly pathogenic at the species level, and were always related to disease. However, from the experimental data collected over recent years, it has become clear that *L. monocytogenes* demonstrates serotype/strain variations in virulence and pathogenicity rate [5]. The population structure of 43 low-virulence strains was investigated with that of 49 virulent strains to estimate their diversity from virulent strains. We also investigated whether low-virulence strains formed a homogeneous subpopulation of *L. monocytogenes* or whether they originated from a random loss of virulence genes and thus diversified in multiple distinct directions.

We based our analysis on PFGE and different DNA-sequence-based approaches. The PFGE gave the greatest discriminatory power. Indeed PFGE gave profiles for different strains that by another way were grouped together in MSTrees. For example, ST2 (Figure 3) comprised low-virulence strains of the phenotypic Groups-I, -V, and -VI, which had different PFGE profiles. Similarly, the low-virulence strains AF105 and LSEA-99-23 exhibited the same MLST profile but had distinct profiles in PFGE. Interestingly, MSTree identified specific ST for half of the low-virulence strains belonging to lineage II.

Overall, we identified low-virulence *L. monocytogenes* strains in both lineages I and II. No hypothesis could be advanced for the lineage III/IV, as they were few strains studied here represented these lineages. Our population structure showed that low-virulence strains are linked firstly according to their lineage, then to their serotypes and after which, they lost their virulence suggesting a relatively recent emergence. MSTree analyses showed that low-virulence strains belonging to lineage II formed a tightly clustered, monophyletic group with limited diversity, in contrast to the low-virulence strains of lineage I. All our observations further supported the fact that some correlations existed between virulence level and point mutations, base substitutions inducing a stop-codon, or inactivation of different virulence proteins, rather than on horizontal transfer or gene loss [7,8,20]. A characteristic of lineage II low-virulence strains was that all strains had a point mutation in the virulence *inlA* gene. Interestingly, there was a strong correlation between the *inlA* mutation and the genotypic group which were based on the mutations responsible for the virulence lost. Moreover, all strains of ST31 had only two different *inlA* mutations, but only the strains with the mutation type 5, according to Van Stelten also have the PrfAK220T mutation [17]. This observation suggested that the *inlA* mutation appeared before the *prfA* mutation. Regardless of the nature of mutations in *inlA* in the different low-virulence strains, there was clearly a link between their prevalence in food environments and the *inlA* mutations. Indeed, the *inlA* mutations were identified mainly in serotypes 1/2a and 1/2c from lineage II isolated from food and food-processing environments [17,21]. As such, it is reasonable to hypothesize that variations within these groups have been shaped to a greater extent by selective constraints operating in food manufacturing-plants.

It is intriguing that InlA, and to a lesser extent PrfA, which are important bacterial factors for host colonization, were lost. This pattern could be explained either by relaxation of the selective constraint to maintain InlA and PrfA function or by a selective advantage provided by the loss of functional virulence proteins in the ecological niche occupied by these strains. Clonal families might be adapted to different niches, and their occurrence as mammalian pathogens may be of limited significance for their evolutionary success in the long term. Considering all altered factors, the low-virulence strains could represent over 50% of the *L. monocytogenes* strains [5]. The fact that the growth of some low-virulence *L. monocytogenes* strains was impaired on selective medium suggests that the prevalence of these strains may be higher than that currently reported [22]. Moreover, only a few *L. monocytogenes* strains isolated from the environment and/or food have been analyzed, in contrast to strains of human origin. Developing reliable and easy-to-perform virulence tests could be useful, particularly for risk analysis, where it is important to evaluate the risk associated with the consumption of food products contaminated with *L. monocytogenes* not only on the basis of levels of bacterial contamination but also on the virulence level of the strains.

In this complex diversity scheme, the case of the A23 strain is very intriguing. Indeed, it is still virulent in mice, despite non-functional major virulence genes, due to point mutations in *inlA*, *inlB* and *plcA* that characterize the genotypic Group-IIIa [15]. This strain was found to be in the same cluster as the Group-IIIa strains using PFGE and MLST analyses, but to be in a specific ST using MSTree (ST 196 and 193, respectively). The fact that this strain has an additional mutation in *mpl* compared to Group-IIIa strains [15] suggests that it evolved from this group and thus reacquired virulence genes after initial virulence-gene loss. However, optical mapping does not support this hypothesis, since compared to the EGDe genome, specific fragments have been inserted in the genome of the Group-IIIa strains but not in strain A23, suggesting that the Group-IIIa strains have evolved from the latter. The complete sequencing of the genome of these strains should clarify this question.

This analysis corroborated the classification obtained for the phenotypic Groups-I and –III. Moreover the new detected low-virulence strains exhibiting the same phenotypes and harbouring the same mutations in the virulence genes, as previously observed, reinforced our observations. The new results allowed us to subdivide the former Group-IV into 3 new Group-IV, -V and –VI and to suggest different hypothesis concerning the population structure and diversity of the low-virulence strains compared to virulent strains.

## Conclusions

The data presented in the present study show indeed that the diversity and population structure according to the virulence level of *L. monocytogenes* strains is complex and based on different mechanisms which seem to differ according to the lineage of the strains and thus to their ecological niches. However, from a practical perspective, this strain population does not correspond to a new species within *Listeria*. The relatively clear differences between virulent and non-virulent strains or species make these bacteria an attractive model for examining the lost of pathogenicity in this genus and for applying these principles to logical predictions of how certain pathogens will behave in a population over evolutionary time.

## Methods

### Strains and culture conditions

The 92 *L. monocytogenes* strains used in this study are described in the Additional file 1. The non-virulent *L. innocua* BUG499 strain was used as negative reference. All isolates were collected from independent sources at different dates. *L. monocytogenes* strains were defined as virulent or low-virulence using a virulence test combining a PF assay performed with the human colon adenocarcinoma cell line HT-29 and subcutaneous inoculation of mice into the hind footpads of immunocompetent Swiss mice as previously described [3]. Animal experiments were carried out in strict accordance with French recommendations. The protocol was approved by the Val de Loire Ethics Committee for Animal Experiments (n° 2011-07-02). For analysis, strains were cultured for 8 h in brain-heart infusion broth (Becton Dickinson, Fisher, Illkirch, France) at 37°C.

The collection of 656 *L. monocytogenes* strains from the French Reference Centre for *Listeria* and the WHO Collaborative Centre for Foodborne Listeriosis were used for the minimum spanning tree (MSTree) (comparative set; Figure 3) as previously described [9,18].

### Phenotypic characterization of the low-virulence strains

The PF assay performed on HT-29 cells and invasion assays performed on Caco-2 and Vero cells were previously described [8]. The detection of the PI-PLC activity assays were analyzed in the culture supernatant with tritium-labelled L-α- phosphatidyl-inositol [8] and the PC-PLC activity was assessed after incubating with lecithin suspension, at 510 nm [7]. Experiments were carried out in duplicate and repeated twice for each strain. The values obtained allowed us to perform an agglomerative hierarchical clustering, based on Ward’s method and the Euclidean distance, to identify groups (clusters).

### Pulsed-Field Gel electrophoresis (PFGE)

The PFGE protocol used in this study was the PulseNet standardized molecular subtyping protocol in accordance with Graves and Swaminathan [23].

The gels were photographed under UV transillumination, and the images were digitized and analyzed using BioNumerics v4.6 software (Applied-Maths, Sint-Martens-Latem, Belgium). The matching of band patterns was based on the DICE coefficient. Dendrograms were created using the Unweighted Pair Group Method with arithmetic mean. Strains were considered to be indistinguishable and were assigned to the same PFGE profile when the dendrogram indicated an index of relatedness of 100% verified by visual examination of band patterns.

### Gene sequencing and multi-locus sequence typing (MLST)

The nucleotide sequencing of *prfA*, *inlA*, *inlB* and *plcA* genes and sequence analyses were described previously [7,8]. The *clpP* gene and its flanking regions (lmo2467 and lmo2469) were amplified from total isolated DNA using PCR. Primers and temperature annealing are listed in the Additional file 2.

The *prfA* and *inlA* virulence genes were fully sequenced, whereas the *actA* gene was partially sequenced. Seven housekeeping genes (*acbZ*, *bglA*, *cat*, *dapE*, *dat*, *ldh*, and *lhkA*) were selected for the MLST analyses (Additional file 2: Table S2) [9]. Alleles and sequence types (ST) are freely available at http://www.pasteur.fr/mlst. For analyses, sequences were concatenated either for the virulence or the housekeeping genes in an MLST scheme. For each MLST locus, including the 748 *L. monocytogenes* strains, an allele number was given to each distinct sequence variant. MLST analysis links profiles so that the sum of the distances (number of distinct alleles between two profiles) is minimized [24]. Each circle represented in Figure 3 corresponds to a ST number, attributed to each distinct combination of alleles on the seven genes. The size of the circle corresponds to the number of strains with that particular profile.

The dendrograms of the concatenated nucleotide sequences of virulence and housekeeping genes with the Neighbor-Joining (NJ) method and MLST analysis were performed using BioNumerics v4.6.

### Optical mapping

Optical maps were prepared on the Argus™ Optical Mapping System by OpGen (Gaithersburg, MD USA), as described previously [25]. This method scans and assesses the architecture of complete bacterial genomes. Briefly, following cell lysis, genomic DNA molecules were spread and immobilized onto derivatized glass slides and digested by *Nco*I. After restriction digestion, a small gap in the DNA at the precise location of the restriction endonuclease cleavage site is left. The DNA digests were stained with YOYO-1 fluorescent dye, and photographed with a fluorescence microscope interfaced with a digital camera. Automated image-analysis software located and sized fragments, based on YOYO-1 binding and assembled multiple scans, into whole-chromosome optical maps. The average size of each restriction fragment (measured in 30–100 different molecules in the assembly) was determined and used to create a linear “consensus map” on which each restriction site is represented by a vertical line.

### Nucleotide sequences

The DNA sequences of the MLST *loci* have been deposited in GenBank under accession numbers EU294615-EU294706 (*abcZ*), EU294707-EU294797 (*bglA*), EU294798-EU294889 (*cat*), EU294890-EU294981 (*dapE*), EU294982-EU295073 (*dat*), (EU295074-EU295165 (*ldh*), EU295166-EU295257 (*lhkA*), EU294523-EU294614 (*prfA*), EU295258-EU295336 (*actA*), and EU295337-EU295423 (*inlA*).

## Competing interests

The authors declare that they have no competing interests.

## Authors’ contributions

OG and ST carried out the molecular genetic studies, participated in the sequence alignment. AK carried out the PFGE analysis. MR and AL carried out the MLST analysis. SMR carried out the phenotypic studies. BS performed the statistical analysis. GK carried out the optical mapping. LM and ALM participated in the design of the study. PhV and SMR conceived of the study, and participated in its design and coordination, helped to draft the manuscript. All authors read and approved the final manuscript.

## Supplementary Material

Additional file 1 **Describes the*Listeria* strains used in this article[**[7]**,**[8]**,**[10]**,**[15]**,**[26-30]**].** Click here for file

Additional file 2 **Describes the primers used for the amplification and sequencing of the housekeeping genes*abcZ*,*bglA*,*dapE*, *dta*, *kat*,*ldh* and *lhkA*and the virulence genes *prfA***, ***actA*and*inlA.*** The primers used for the verification of an inserted fragment in the “*clpP*” region have been also given. Click here for file
