# Supplementary material for: Polyphasic characterization and genetic relatedness of low-virulence and virulent Listeria monocytogenes isolates
Source: BMC Microbiol. 2012 Dec 26;12:304. doi: 10.1186/1471-2180-12-304 (PMC3558321; doi:10.1186/1471-2180-12-304)
Supplement: Additional file 1 — Describes theListeria strains used in this article[[7],[8],[10],[15],[26-30]]. [file 1471-2180-12-304-S1.doc]

**Additional file 1**. *Listeria* strains used

| **Low-virulence strains** | **Origin** | **Serotype** | **Source** | **References** |
| --- | --- | --- | --- | --- |
| CHU 860776 | 1 | 1/2 a | Food product | [7] |
| CNL 895793 | 1 | 1/2 a | Food product | [7] |
| CNL 895803 | 1 | 1/2 a | Food product | [7] |
| CNL 895804 | 1 | 1/2 a | Food product | [7] |
| CNL 895806 | 1 | 1/2 a | Food product | [7] |
| CNL 895809 | 1 | 1/2 a | Food product | [7] |
| SO49 | 2 | 1/2 a | Food product | [7] |
| AF10 | 3 | 1/2 a | Food product | [7] |
| BO18 | 2 | 1/2 a | Food product | [7] |
| BO38 | 2 | 1/2 a | Food-manufactoring plant | [7] |
| AF95 | 3 | 1/2 a | Food-manufactoring plant | [7] |
| CNL 895807 | 4 | 1/2 a | Food product | [8] |
| CNL 895795 | 1 | 1/2 a | Food product | [8] |
| 416 | 1 | 1/2 a | Food product | [8] |
| 417 | 1 | 1/2 a | Food product | [8] |
| BO43 | 2 | 1/2 a | Food product | [8] |
| DSS794AA1 | 5 | 1/2 a | Food product | this study |
| DSS1130BFA2 | 5 | 1/2 a | Food product | this study |
| DPF234HG2 | 5 | 1/2 a | Food product | this study |
| LSEA 99-4 | 6 | 1/2 a | Sludge | this study |
| AF105 | 3 | 1/2 c | Food-manufactoring plant | [7] |
| LSEA 99-23 | 6 | 3c | Activated sludge | [25] |
| 99EB15LM | 3 | 1/2 a | Food-manufactoring plant | this study |
| 99EB24LM | 3 | 1/2 a | Food product | this study |
| 99EB04LM | 3 | 1/2 a | Food product | this study |
| 454 | 1 | 4d ou 4e | Food product | [7] |
| 436 | 1 | 4b | Food product | [7] |
| 442 | 1 | 4d ou 4e | Food product | [7] |
| 449 | 1 | 4b | Food product | [7] |
| 464 | 1 | 4b | Human clinical case | [7] |
| BO34 | 2 | 4b | Food product | [7] |
| CR282 | 4 | 7 | Human clinical case | [7] |
| 09-98 SRV 10a Al1 | 7 | 4d | Environmental strain | this study |
| 09-98 SRV 10b Al2 | 7 | 4b | Environmental strain | this study |
| 02-99 SLQ 10c Al | 7 | 4b | Environmental strain | this study |
| 04-99 EBS 1lb Al | 7 | 4b | Environmental strain | this study |
| 11-99 SRV 1a Al | 7 | 4d | Environmental strain | this study |
| NP 26 | 8 | 4b | Food product | this study |
| 09-98 HPR 50a Al1 | 7 | 4d | Food product | this study |
| 3876 | 9 | 4b ou 4d ou 4e | Food product | this study |
| 3877 | 9 | 4b ou 4d ou 4e | Food product | this study |
| N2 | 1 | 4 | Animal clinical case | this study |
| LSEA 00-14 | 6 | 4b | Dewatered sludge | [25] |
|  |  |  |  |  |
| **Virulent strains** |  |  |  |  |
| EGDe BUG 1600 | 4 | 1/2 a | Animal clinical case | [26] |
| A23 | 1 | 1/2 a | Food product | [14] |
| H3 | 1 | 1/2 a | Epidemic | this study |
| H20 | 1 | 1/2 a | Human clinical case | this study |
| H35 | 1 | 1/2 a | Human clinical case | this study |
| A3 | 1 | 1/2 a | Food product | this study |
| E4 | 1 | 1/2 a | Food-manufactoring plant | this study |
| N15 | 1 | 1/2 a | Animal clinical case | this study |
| A24 | 1 | 1/2 a | Food product | this study |
| N13 | 1 | 1/2 a | Animal clinical case | this study |
| LO28 | 10 | 1/2 c | Human isolate | [27] |
| AF104 | 3 | 1/2 c | Food-manufactoring plant | this study |
| AF30 | 3 | 3c | Food product | this study |
| 96102 | 11 | 1/2 a | Animal clinical case | this study |
| 99011 | 11 | 1/2 a | Animal isolate | this study |
| 429 | 1 | 1/2 a | Human clinical case | this study |
| 423 | 1 | 1/2 a | Human clinical case | this study |
| AF39 | 3 | 1/2 a | Food-manufactoring plant | this study |
| AF114 | 3 | 1/2 a | Food-manufactoring plant | this study |
| AF12 | 3 | 1/2 a | Food product | this study |
| AF143 | 3 | 1/2 a | Food-manufactoring plant | this study |
| 12-98 HLQ 5c Al | 7 | 1/2 a | Food product | this study |
| 02-99 ERV 1la Al | 7 | 1/2 a | Environmental strain | this study |
| LSEA 00-24 | 6 | 1/2 a | Stored sludge | [25] |
| 11-98 SIB 10c Al | 7 | 1/2 a | Environmental strain | this study |
| H27 | 1 | 4a | Human clinical case | [28] |
| ATCC 19114 | 12 | 4a | Animal clinical case | [29] |
| N19 | 1 | 4a | Animal clinical case | this study |
| ATCC 19116 | 12 | 4c | Animal isolate | [29] |
| A13 | 1 | 4b | Food product | this study |
| A25 | 1 | 1/2 b | Food product | this study |
| H67 | 1 | 1/2 b | Human clinical case | this study |
| H16 | 1 | 4b | Human clinical case | this study |
| A8 | 1 | 1/2 b | Food product | this study |
| H52 | 1 | 3b | Human clinical case | this study |
| H5 | 1 | 4b | Human clinical case | this study |
| 96031 | 11 | 4b | Animal isolate | this study |
| 98034 | 11 | 4b | Environmental strain | this study |
| epid France 69 | 1 | 4b | Epidemic | this study |
| epid Suisse 55 | 1 | 4b | Epidemic | this study |
| epid GB 38 | 1 | 4b | Epidemic | [30] |
| AF28 | 3 | 4b | Food product | [7] |
| ATCC 19115 | 12 | 4b | Human clinical case | [29] |
| AF40 | 3 | 4b | Food product | this study |
| AF90 | 3 | 4b | Animal isolate | this study |
| 96015 | 11 | 1/2 b | Feed product | this study |
| 97082 | 11 | 1/2 b | Feed product | this study |
| 98066 | 11 | 1/2 b | Feed product | this study |
| AF160 | 3 | 1/2 b | Food product | this study |

All strains originating from different countries and sources, over many years.

^1^ Strains originating from Faculté de Médecine, unité EA 2105 (Tours, France)

^2^ Strains originating from Soredab (La Boissière-Ecole, France)

^3^ Strains originating from Agence Nationale de Sécurité Sanitaire de l'Alimentation, de l'Environnement et du Travail (Maisons-Alfort, France)

^4^ Strains originating from Institut Pasteur, Centre National de Référence des *Listeria* (Paris, France)

^5^ Strains originating from Agence Nationale de Sécurité Sanitaire de l'Alimentation, de l'Environnement et du Travail (Boulogne sur Mer, France)

^6^ Strains originating from Laboratoire des Sciences de l’Environnement et de l’Aménagement (Angers, France)

^7^ Strains originating from Université de Rennes (Rennes, France)

^8^  Strain originating from Institut de Recherche Laitière et de Bactériologie (Vienne, Autriche)

^9^ Strains originating from Institut Fédéral pour la Protection de la Santé des Consommateurs et Médecine Vétérinaire (Berlin, Allemagne)

^10^ Strain originating from Faculté de Médecine Necker-Enfants Malades (Paris, France)

^11^ Strains originating from Institut National de la Recherche Agronomique (Nouzilly, France)

^12^ Strains originating from American Type Culture Collection (Rockville, MD, USA
