# Supplementary material for: Polyphasic characterization and genetic relatedness of low-virulence and virulent Listeria monocytogenes isolates
Source: BMC Microbiol. 2012 Dec 26;12:304. doi: 10.1186/1471-2180-12-304 (PMC3558321; doi:10.1186/1471-2180-12-304)
Supplement: Additional file 2 — Describes the primers used for the amplification and sequencing of the housekeeping genesabcZ,bglA,dapE, dta, kat,ldh and lhkAand the virulence genes prfA, actAandinlA. The primers used for the verification of an inserted fragment in the “clpP” region have been also given. [file 1471-2180-12-304-S2.doc]

**Additional file 2**. Primers used in this study

1. Amplification and sequencing of housekeeping genes

| Gene | Primers (5’→ 3’) | Gene location (a) | Annealing temperature (ºC) |
| --- | --- | --- | --- |
| *abcZ* | fwd – GTTTTCCCAGTCACGACGTTGTATCGCTGCTGCCAC  TTTTATCCA | 2828236 to 2830008 | 52 |
|  | rev - TTGTGAGCGGATAACAATTTCTCAAGGTCGCCGTTTAGAG |  |  |
| *bglA* | fwd – GTTTTCCCAGTCACGACGTTGTAGCCGACTTTTTATGGGGTGGAG | 343221 to 344636 | 45 |
|  | rev – TTGTGAGCGGATAACAATTTCCGATTAAATACGGTGCGGACATA |  |  |
| *dapE* | fwd – GTTTTCCCAGTCACGACGTTGTACGACTAATGGGCATGAAGAACAAG | 287853 to 288992 | 52 |
|  | rev – TTGTGAGCGGATAACAATTTCATCGAACTATGGGCATTTTTACC |  |  |
| *dat* | fwd – GTTTTCCCAGTCACGACGTTGTAGAAAGAGAAGATGCC  ACAGTTGA | 1661588 to 1662457 | 52 |
|  | rev – TTGTGAGCGGATAACAATTTCTGCGTCCATAATACACCATCTTT |  |  |
| *kat* | fwd – GTTTTCCCAGTCACGACGTTGTAATTGGCGCATTTTGAT  AGAGA | 2871318 to 2872784 | 52 |
|  | rev – TTGTGAGCGGATAACAATTTCAGATTGACGATTCCTGCTTTTG |  |  |
| *ldh* | fwd – GTTTTCCCAGTCACGACGTTGTAGTATGATTGACATAGATAAAGA | 214486 to 215427 | 50 |
|  | rev – TTGTGAGCGGATAACAATTTCTATAAATGTCGTTCATACCAT |  |  |
| *lhkA* | fwd – GTTTTCCCAGTCACGACGTTGTAAGAATGCCAACGACG  AAACC | 1538498 to 1539937 | 52 |
|  | rev – TTGTGAGCGGATAACAATTTCTGGGAAACATCAGCAA  TAAAC |  |  |
| Sequencing primers | Fwd - GTT TTC CCA GTC ACG ACG TTG T |  |  |
|  | Rev - TTG TGA GCG GAT AAC AAT TTC |  |  |

1. Amplification and sequencing of virulence genes

| Gene | Primers (5’→ 3’) | Gene location (a) | Annealing temperature (ºC) |
| --- | --- | --- | --- |
| *prfA* | prfA1 - GCTAACAATTGTTGTTACTGCC | 203640 to 204353 | 60 |
|  | prfA2 - GAAGCAATCGTACGCGTTCAT |  |  |
|  |  |  |  |
| *inlA* | inlA1 - CATGATTTTTCGGATGCAGGAG | 454534 to 456936 | 60 |
|  | inlA2 - TTTGTTAGACCCGACAGTGGTG |  |  |
|  |  |  |  |
|  | inlA3 - AGTCTTATCGCTACTAACAACC |  | 60 |
|  | inlA4 - TCCATTTAGTTCCGCCTGTTTG |  |  |
|  |  |  |  |
|  | inlA5 - AACATTTAGTGGAACCGTGACG |  | 56 |
|  | inlA6 - ACTATCCTCTCCTTGATTCTAG |  |  |
|  |  |  |  |
| *actA* | actA1 - TCCAACAGAAGATGAACTAG | 209470 to 211389 | 55 |
|  | actA2 - TTTGGATTACTGGTAGGCTCGG |  |  |

**C.** Verification of an inserted fragment in the “*clpP*” region

| Gene | Primers (5’→ 3’) | Gene location (a) | Annealing temperature (°C) |
| --- | --- | --- | --- |
| *clpP* | fwd - TGACCTAGTTTGACCATTCGTG | 2542619 to 2542656 | 55 |
| (lmo2468) | rev - GAAGAAAACATTCAGCATTGCG |
|  |  |  |  |
| lmo2467 | début - TCCAATCTTCGTCATTTTCTTC | 2540421 to 2541857 | 55 |
|  | fin - TGGATGAACTATCAGGCTTCTC |
|  |  |  |  |
| lmo2469 | début-CATAACCCAGCGACTGATAAAC | 2542658 to 2544049 | 55 |

(a) Positions correspond to complete genome sequence of strain EGDe (NC003210)
